# An immunoturbidimetric assay for bovine haptoglobin

**DOI:** 10.1007/s00580-018-2863-6

**Published:** 2018-11-14

**Authors:** Nicola Brady, Emily L. O’Reilly, Christopher McComb, Alastair I. Macrae, P. David Eckersall

**Affiliations:** 1 Institute of Biodiversity, Animal Health and Comparative Medicine, College of Medical, Veterinary and Life Sciences, University of Glasgow, Glasgow, G41 4HQ UK; 2 Present Address: Biomedical Teaching Organisation, University of Edinburgh Medical School, Teviot Place, Edinburgh, EH8 9AG UK; 3 Dairy Herd Health and Productivity Service, Royal (Dick) School of Veterinary Studies and the Roslin Institute, Easter Bush Veterinary Centre, University of Edinburgh, Roslin, Midlothian, EH25 9RG UK; 4 Present Address: ERA Chair Laboratory, VetMedZg, Faculty of Veterinary Medicine, University of Zagreb, Heinzelova 55, 10000 Zagreb, Croatia

**Keywords:** Acute phase, Haptoglobin, Cattle, Immunoturbidimetric

## Abstract

In cattle, the serum protein haptoglobin (Hp) is a major acute phase protein (APP) that rises in concentration over a thousand fold following stimulation by pro-inflammatory cytokines. As such, this APP is a valuable biomarker for infection, inflammation and trauma in cattle. The assay for bovine Hp is becoming more commonplace in clinical pathology and in experimental studies when a biomarker of innate immunity is required. The most widely used assay for Hp utilises its binding to haemoglobin (Hp-Hb binding assay), which at low pH enables the preservation of the native peroxidase activity in the haemoglobin. This assay is used for all species, including species such as dog, cat and pig where the level of Hp is higher in healthy animals of these species than in healthy cattle, and therefore a bovine-specific immunoassay that can be automated would be desirable. Thus, a novel-automated species-specific immunoturbidimetric (IT) assay has been developed. Validation studies showed intra- and inter-assay CVs of below 5% and 9% respectively and a recovery of 99% from samples spiked with bovine Hp and a limit of quantification of 0.033 g/L. The assay is not affected by icterus or lipaemia but had moderate interference from haemoglobin and showed a significant correlation with the Hp-Hb binding assay. This novel IT assay for bovine Hp will allow automated analysis of this important bovine APP to identify changes in the Hp concentration not detectable by current Hp-Hb binding assays. It will enable the incorporation of this assay into herd health assessments, animal welfare analysis and for bovine medicine and research.

## Introduction

Haptoglobin (Hp) is a serum protein that in many species is an acute phase protein (APP), increasing in concentration during innate immune responses to infection, inflammation and trauma (Ceron et al. 2005; Cray et al. 2009; Eckersall and Bell 2010). Acute phase proteins are stimulated by pro-inflammatory cytokines such as interleukin (IL)-1, IL-6 and tumour necrosis factor-α (Moshage 1997). It has been demonstrated that measurement of the circulating concentration of Hp provides valuable diagnostic information on the host’s innate immune responses with over 1000-fold increases occurring in some species such as cattle and sheep where it is classified as a major APP (Ceciliani et al. 2012; Horadagoda et al. 1999). Haptoglobin is also a valuable serum biomarker in species such as dogs, pigs and human where increases may only be up to 10-fold the resting levels, where it is classified as a moderate APP. As a biomarker of innate immune responses, the increased serum concentrations of APP reflect active disease processes and, although they are not specific for particular diseases, they are highly sensitive indicators of pathological processes following infection or inflammation across veterinary species (O’Reilly and Eckersall 2014; Schmidt and Eckersall 2015). Thus, over recent decades, the measurement of serum Hp in a variety of species has become more common, and there have been extensive reports of the use of measuring this APP in investigations of both experimental and clinical studies (Eckersall and Bell 2010). In cattle, raised serum Hp has been demonstrated in diseases of respiratory, reproductive, digestive and mammary functions and has also been identified as a biomarker of peri-parturient disease (Ceciliani et al. 2012).

The analysis of serum Hp for analysis in comparative clinical pathology was facilitated by the development of a biochemical assay based on the biological activity of Hp binding to haemoglobin (Hp-Hb binding assay) (Eckersall et al. 1999). This assay format led to the automation of the assay on biochemical analysers which are located in most veterinary diagnostic laboratories. While the Hp-Hb binding assay is a valuable diagnostic test to use on a biochemical analyser and can detect and quantify increased levels of Hp in species where it undergoes an APP response, there are problems inherent when measured on certain biochemical analysers that can lead to reduced precision. The Hp-Hb binding assay was developed for analysers which operate with three reagents and the Hp-Hb binding assays have three reagents, namely (i) a solution of haemoglobin, (ii) a chromogen, which is a peroxidase substrate in buffer and (iii) peroxide. In recent years, many biochemical analysers are used in veterinary laboratories which accommodate only two reagents. The Hp-Hb binding assay can only be used on these analysers if two of the reagents are mixed, such as a mix of the chromogen (containing a peroxide substrate) and peroxide, or a mix of peroxide and haemoglobin which has an innate peroxidase activity. Both of these mixtures are inherently unstable and have a short shelf life so if not used immediately can cause poor precision.

To measure proteins in human serum in clinical biochemistry laboratories, at similar concentrations as found in bovine serum for Hp, the method of choice is by immunoturbidimetric (IT) assays (Eckersall et al. 1991; Ledue et al. 2002; Price et al. 1983). These are immunoassays based on antibody-antigen interaction and are rapid as well as being specific and sensitive and only require two reagents being assay buffer and an antibody solution. Therefore, the aim of this study was to develop an IT assay for bovine Hp, based on species-specific antiserum that can be used in all biochemical analysers, including those that are designed to only use two reagents, and to compare its function to an established Hp-Hb binding assay (Eckersall et al. 1999).

## Materials and methods

Antiserum to bovine haptoglobin (Life Diagnostics Inc., West Chester, USA) raised in sheep (Diagnostic Scotland, Edinburgh, UK) was used in the development of the IT assay. The IT assay was set up on an ABX Pentra 400 automated biochemical analyser (HORIBA UK Ltd., Northampton, UK), initially using parameters based on those previously described for canine C-reactive protein (Eckersall et al. 1991) using a pool of bovine serum with high Hp as standard, which was calibrated against the International Reference Preparations of bovine serum archived at the University of Copenhagen (Prof. P. Heegaard). Standards were prepared by dilution of the pool of high Hp (1.49 g/L), to give further Hp concentrations of 1.18, 0.57, 0.027 and 0.00 g/L. The diluent was ovine serum in order to minimise matrix effects caused by the use of buffer alone as diluent. The optimised assay protocol consisted of 5 μL of sample or standard added to 150 μL of 4% (*w*/*v*) polyethylene glycol 8000 (Sigma) in 20 mM pH8.0 Tris buffered saline (TBS) with 1.0% (w/v) Tween 20, and 10 μL distilled water, followed by the addition of 35 μL of sheep anti-bovine Hp antiserum at 48 s and run for a total of 300 s with absorbance being monitored at 340 nm. The concentration of Hp in samples was determined against the standard curve using a 4-parameter fit model in the integrated computer system of the biochemical analyser.

Laboratory validation of the IT Hp assay was performed to determine specificity, precision, accuracy, limit of detection and interference following Hillstrom et al. (2014) and Piñeiro et al. (2018). The specificity of the antiserum was assessed by Ouchterlony agarose gel immunodiffusion in a 1% (*w*/*v*) agarose gel prepared in 20 mM pH 7.4 TBS. The centre well contained sheep anti-bovine Hp antiserum as used in the IT assay. Commercially available purified bovine haptoglobin with a Hp concentration of 0.84 g/L (Life Diagnostics Inc., West Chester, USA) and bovine serum samples with different Hp concentrations (1.49, 1.18, 0.57, 0.027 and 0.00 g/L), as determined by the Hp-Hb binding assay, were placed in the outer wells.

The precision of the assay was determined by analysing low, medium and high Hp serum samples six times each in the same assay run to determine the intra-assay coefficient of variance (CV). For the inter-assay CV low, medium and high Hp serum samples were run individually over six consecutive days.

Accuracy was assessed by spiking the zero standard with commercially available purified bovine haptoglobin (Life Diagnostics Inc. West Chester, USA). This was serially diluted 1:2, 1:4, 1:8 and 1:16, and each dilution was measured in triplicate. The percentage recovery was calculated for each dilution. A low and a high Hp serum sample were diluted 1:2 and 1:4 to determine the linearity of the assay and parallelism to the standard curve. Each dilution was measured in triplicate.

To determine operation of the IT assay with low concentrations of Hp the approaches described by Hillstrom et al. (2014) and Piñeiro et al. (2018) were used. The zero standard (ovine serum) was measured 12 times in a single run in order to calculate the limit of blank, calculated as the mean of the zero standard (*μ*_b_) plus three standard deviations of the blank (*σ*_b_). The Limit of Detection was determined by measuring the standard deviation of replicates (*n* = 8) of a sample with low concentration of Hp (*σ*_s_). The Limit of Detection (LoD) was calculated (Piñeiro et al. 2018) as

$$ \mathrm{LoD}={\mu}_{\mathrm{b}}+1.645{\sigma}_{\mathrm{b}}+1.645{\sigma}_{\mathrm{s}} $$

The Limit of Quantitation (LoQ) was determined by measuring the Hp in replicates of dilutions of a low Hp bovine sample (1:8, *n* = 8; 1:16, *n* = 15 over 2 runs; 1:32, *n* = 14 over 2 runs, 1:64 *n* = 1) and was the lowest concentration when the total error (TE) was lower than the allowable total error (TE_a_) set at 29.6% (Hillstrom et al. 2014).

For interference studies, a stock solution containing 30 g/L of haemoglobin (Eckersall et al. 1999) was used to spike a high Hp serum sample with increasing concentrations of haemoglobin (0.031 to 4 g/L). To determine the interference from bilirubin, the metabolite was added to bovine serum with a high level of Hp, at concentrations of bilirubin from 1.56 to 50 mM. To determine the effect of lipaemia, Intralipid (Sigma Chem Co, Poole, UK) was used to spike a high Hp serum sample to give samples with a final triglyceride concentration of 1.08–50 g/L respectively. Each sample for the interference studies was measured in triplicate.

For comparison to an established Hp-Hb binding assay (Eckersall et al. 1999), bovine serum samples (*n* = 182) were analysed for Hp with results from both assays compared. The Hp-Hb binding method was performed as described by Eckersall et al. (1999) with minor modifications. The chromogen reagent was altered by use of an alternative peroxidase substrate, SAT-3 an *o*-tolidine analogue, which after oxidation has an absorbance maximum at 660 nm, by replacement of dithioerythritol with cysteine as a reducing agent and by using triclosan as an anti-bacterial agent. The haemoglobin solution and peroxide reagent remained the same as previously described (Eckersall et al. 1999).

The final working concentrations in the reagents were as follows. Hb reagent was 60 mg/L of Hb 0.154 M NaCl. The chromogen reagent was prepared in 60 mM citric acid and 100 mM di-sodium phosphate at pH 3.8 containing 20 mM phenol, 3.6 mM l-cysteine, 0.32 mM SAT-3 (NBS Biologicals Ltd., Cambridge, UK, Dojindo Inc., Japan), 1% (*v*/*v*) Tween 20 and 0.001% *w*/*v* triclosan. The substrate peroxide reagent was 0.12% (*v*/*v*) H_2_O_2_ diluted from a stock of 30% H_2_O_2_. Chemicals were obtained from Sigma-Aldrich Co Ltd., Poole, UK, unless otherwise stated.

The assay was performed on the ABX Pentra 400 (Horiba UK Ltd., Northampton, UK) automated biochemical analyser with incubation at 37 °C and was calibrated with a pool of porcine serum with Hp concentration of 1.4 g/L determined by comparison to the International Reference Preparations of bovine serum from the University of Copenhagen (Prof. P. Heegaard). Sample or standard (3 μL) was mixed with 100 μL of Hb reagent and after mixing, 50 μL of chromogen reagent was added. After further mixing, 40 μL of peroxide reagent was added and the absorbance was measured after 5 min at 660 nm. Standards of Hp at 1.4, 0.7, 0.35 and 0 g/L were run in each assay and used for calculation of the Hp concentration in each sample using a four-parameter fit model on the computer system of the biochemical analyser.

For comparison between this established assay and the novel IT, serum samples were run in both assays using residual bovine serum samples collected from adult dairy cows by the University of Edinburgh Royal (Dick) School of Veterinary Studies Langhill dairy farm (UK), collected as part of their annual Johnes Disease accreditation test. Their use in this study followed ethical approval by the Royal (Dick) School of Veterinary Studies Veterinary Ethical Review Committee Ref 32-17. The comparison between assays was initially performed on samples (*n* = 108) that had a Hp concentration above the limit of quantification of the Hp-Hb binding assay of 0.03 g/L.

Statistical analysis was performed using Graphpad Prism version 6 (Graphpad Software Inc. CA, USA) to calculate mean, SD and CV, for regression analysis and the Bland-Altman analysis.

## Results

**Fig. 1:**
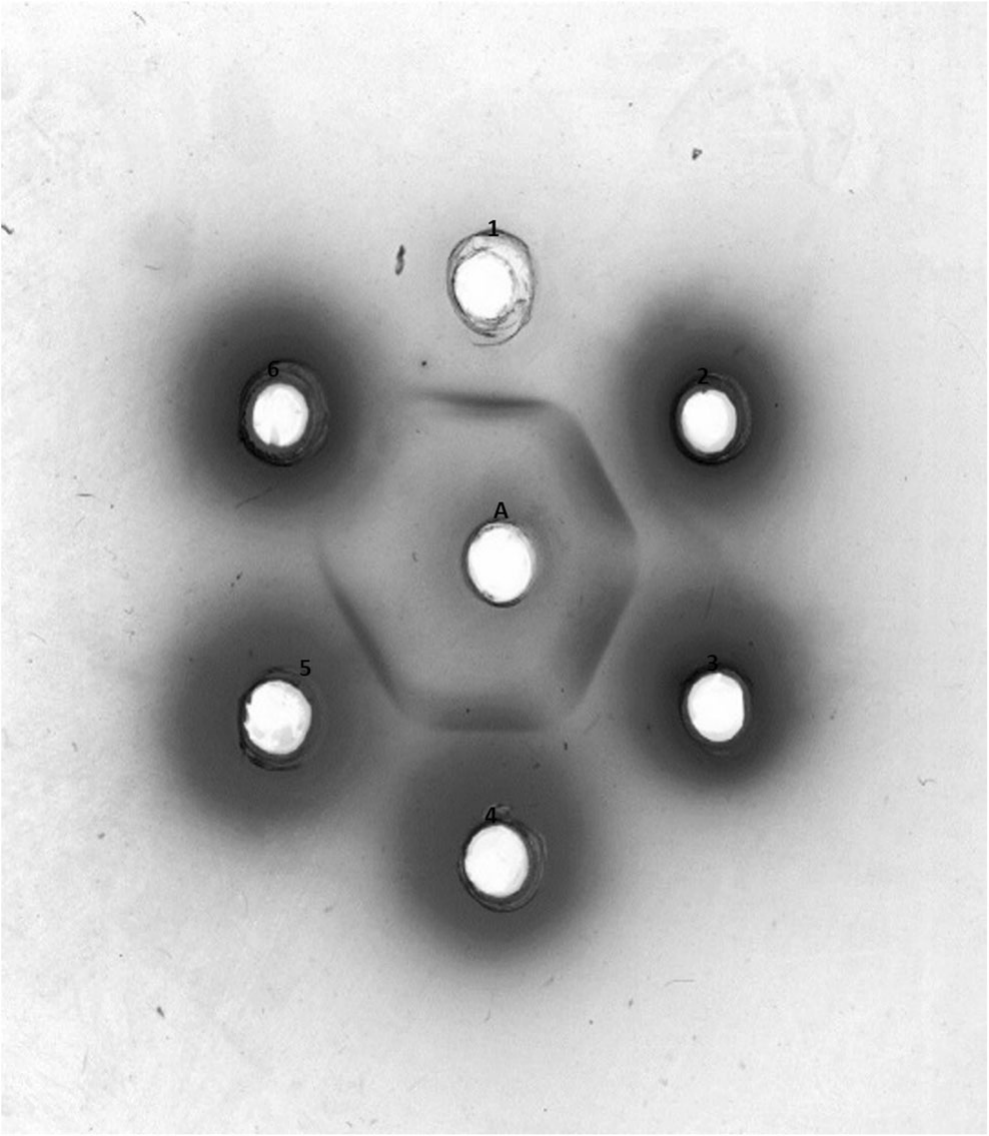
Immunodiffusion in agarose gel. Centre well contained: A: sheep anti-bovine antiserum. Outer wells contained 1: purified bovine Hp at 0.84 g/L; 2: bovine serum containing 1.49 g/L of Hp; 3: bovine serum containing 1.18 g/L of Hp; 4: bovine serum containing 0.57 g/L of Hp; 5: bovine serum containing 0.27 g/L of Hp; 6: bovine serum containing 0.00 g/L of Hp

The specificity of the assay depends on the reactivity of the antiserum to bovine Hp, which was determined in the immunodiffusion gel shown in Fig. 1. Bands of precipitation were detected when sheep anti-bovine Hp antiserum reacted with purified bovine Hp and bovine serum samples containing Hp. The continuous lines of precipitation indicate that the antigen in the bovine serum samples being measured was bovine Hp. No precipitation was observed around the wells containing bovine serum with undetectable Hp.

The precision of the Hp IT assay is given in Tables 1 and 2, with intra-assay CV of 4.1% and 5.0% at low and high QC sample. Inter-assay CVs were 7.4% for a low QC sample and 8.1% for the high QC sample.

**Table 1:** Intra-assay precision (*n* = 6) of immunoturbidimetric assay for bovine haptoglobin

QC sample	Mean (g/L)	SD	CV %
Low QC	0.21	0.009	4.1
High QC	1.08	0.54	5.0

**Table 2:** Inter-assay precision (*n* = 6) of immunoturbidimetric assay for bovine haptoglobin

QC sample	Mean (g/L)	SD	CV %
Low QC	0.15	0.011	7.4
High QC	1.09	0.088	8.1

**Fig. 2:**
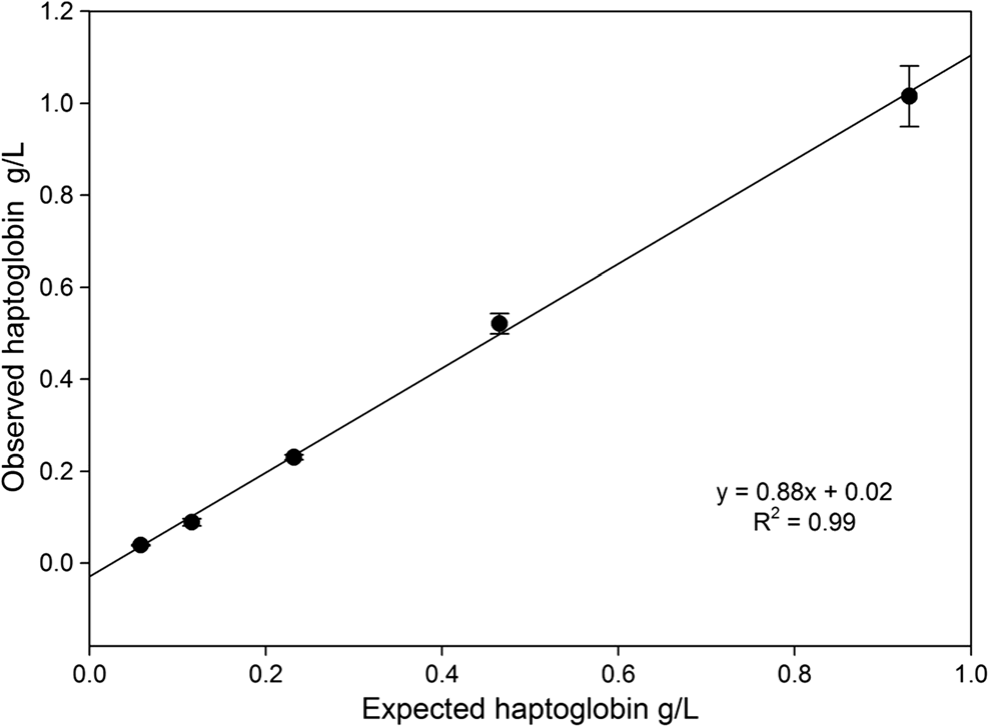
Accuracy determined by spiking Hp to zero standard and assayed with the immunoturbidimetric assay for haptoglobin

The accuracy of the Hp IT assay determined by recovery of added Hp to a sample serum with an undetectable concentration gave a mean recovery of 99% (Table 3), ranging from 76% at the expected value of 0.12 g/L to 112% at an expected 0.47 g/L. The recovery is depicted in Fig. 2, with the relation between observed and expected Hp concentrations giving a correlation coefficient (*R*^2^) of 0.99.

**Table 3:** Recovery of haptoglobin added to blank sample (*n* = 4)

Expected Hp (g/L)	Observed Hp (g/L) Mean ± SD	Recovery (%)
0.93	1.02 ± 0.07	109
0.47	0.52 ± 0.02	112
0.23	0.23 ± 0.005	99
0.12	0.09 ± 0.001	76
	Average =	99

**Fig. 3:**
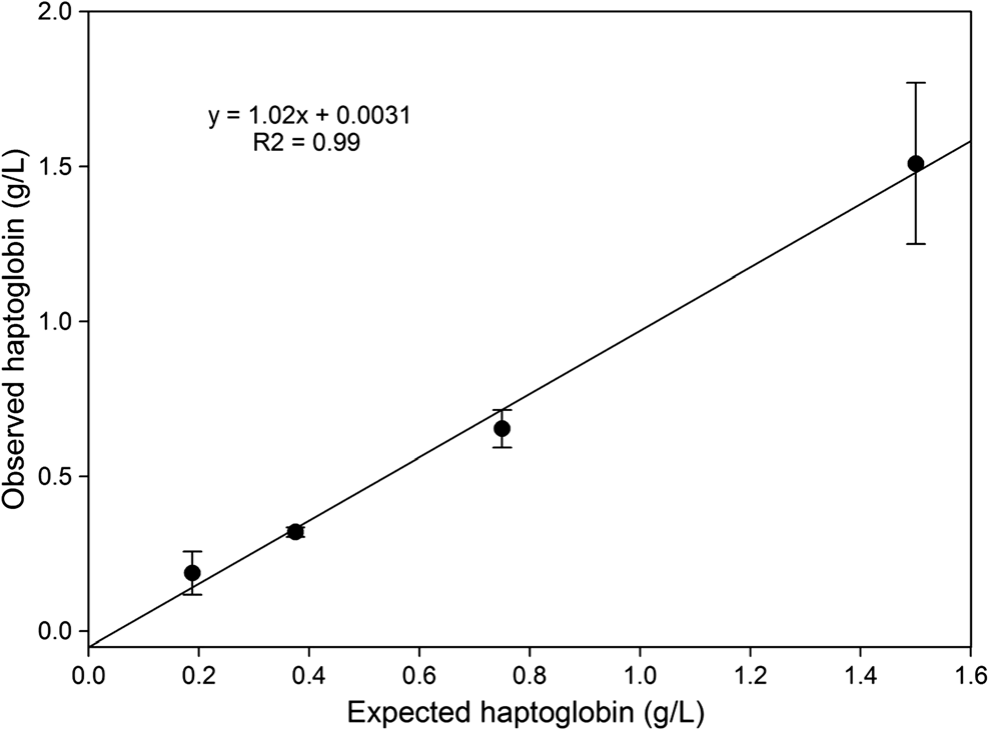
Linearity determined by dilution of a bovine serum with a high-concentration haptoglobin and serially diluted and then assayed by the immunoturbidimetric assay for haptoglobin

The linearity of the dilution of a sample of high Hp concentration diluted in a serum of negligible Hp concentration showed the dilution curve given in Fig. 3, with a correlation coefficient (*R*^2^) of 0.99 and a regression equation of Observed Hp (*y*) = 1.02 Expected Hp (*x*) + 0.0031.

**Fig. 4:**
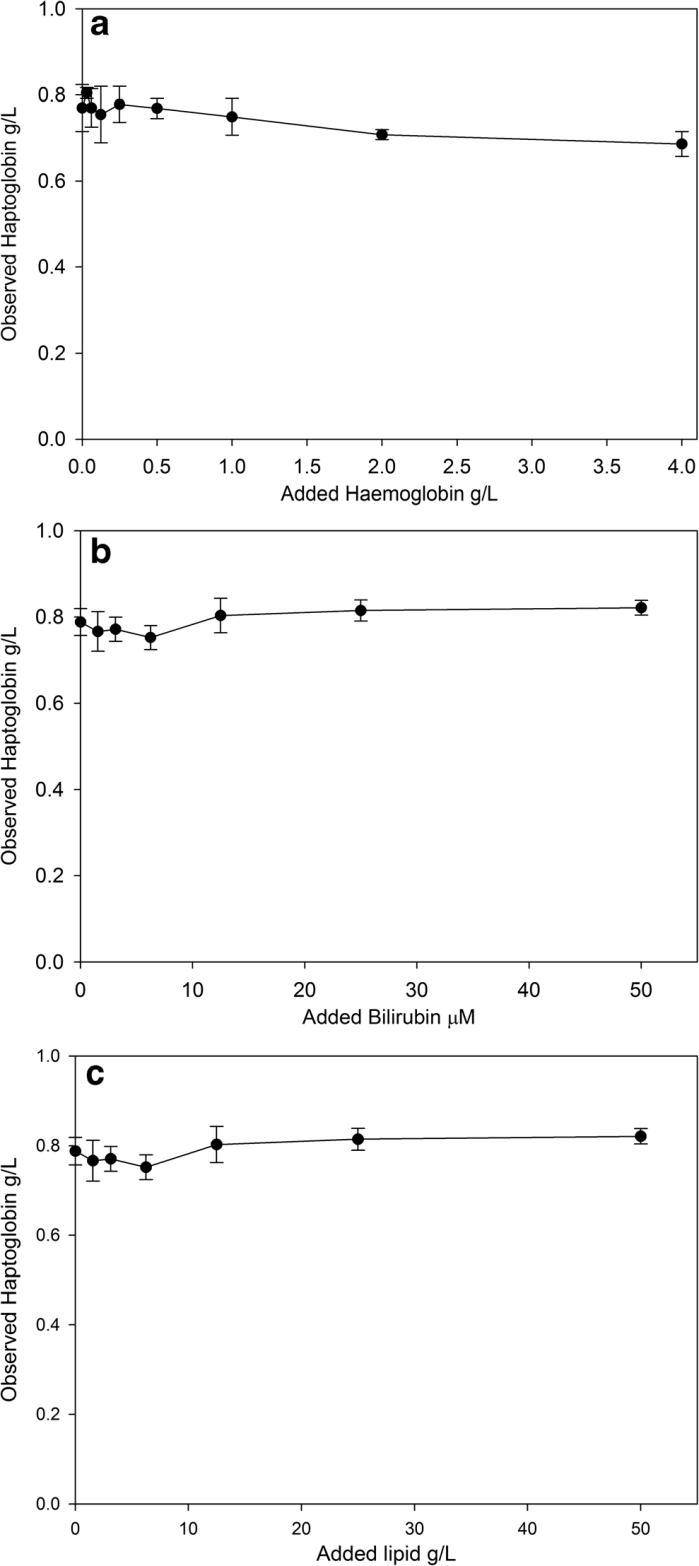
(**a** Top panel) The effect of free haemoglobin on the immunoturbidimetric assay for haptoglobin. (**b** Middle panel) The effect of bilirubin on the immunoturbidimetric assay for haptoglobin. (**c** Bottom panel) The effect of lipaemia on the immunoturbidimetric assay for haptoglobin

The interference in the Hp IT assay caused by Hb showed there was no effect up to a level of 0.5 g/L of Hb in the serum, with a gradual effect thereafter with a 10% reduction in the Hp result at a level of 4 g/L of Hb in the serum (Fig. 4a). Bilirubin had a minimal effect on the IT Hp assay up to a concentration of 50 mM (Fig. 4b). Lipaemia did not cause any change in the measured Hp concentration (Fig. 4c). The limit of blank of the Hp IT assay was calculated to be 0.011 g/L being the Hp concentration at 3 SD from the mean of the blank sample, the limit of detection was 0.018 g/L and the limit of quantification was 0.033 g/L (Table 4).

**Table 4:** Limit of quantification determined as the lowest concentration of Hp in a serially diluted, low Hp sample where the total error (TE) was less than allowable total error (TE_a_) of 26.9%

Expected Hp concentration (g/L)	Mean observed Hp concentration (g/L)	SD (g/L)	TE (%)	TE < TE_a_
0.133	0.136	0.0073	12.8	Yes
0.066	0.059	0.0084	26.2	Yes
0.033	0.028	0.0054	25.4	Yes
0.016	0.047	–	193*	No

*TE=Bias as not repeated to conserve antiserum reagent

**Fig. 5:**
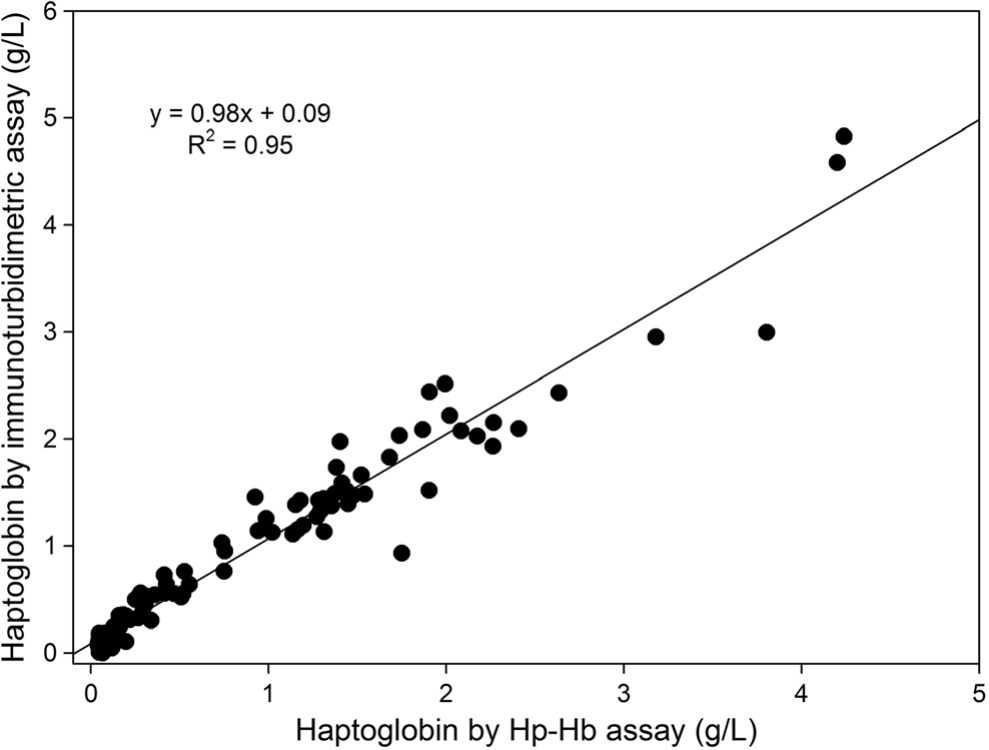
Correlation of bovine Hp estimated by immunoturbidimetric assay with bovine Hp estimated by Hp-Hb binding assay (*n* = 108)

**Fig. 6:**
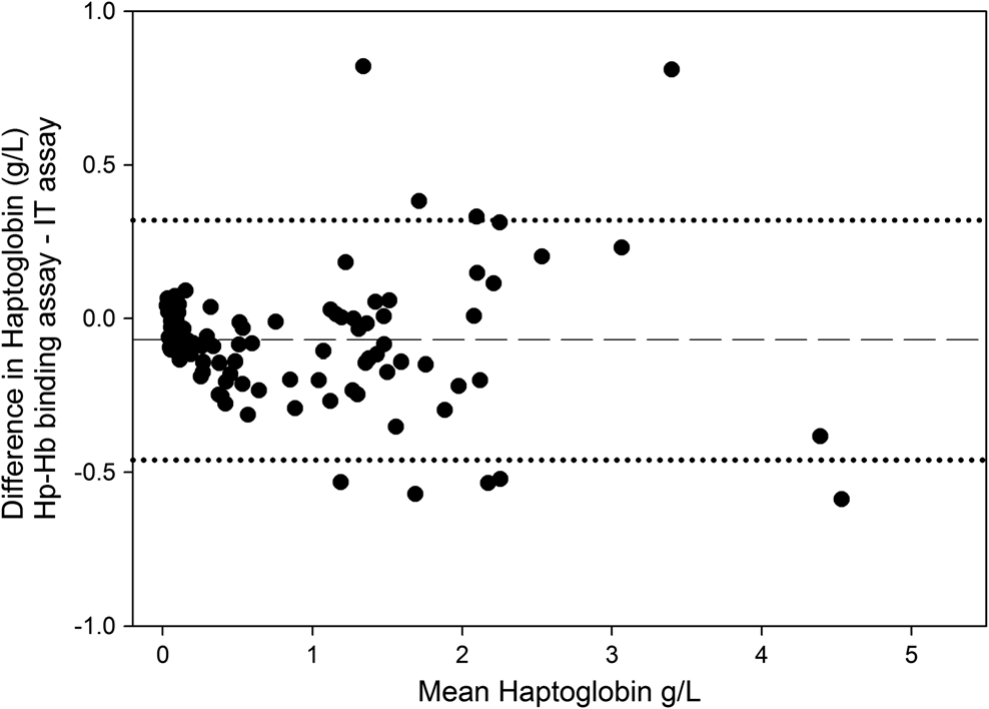
Bland-Altman difference plot for Hp concentrations in bovine serum/plasma samples measured with immunoturbidimetric method and Hp = Hb binding assay (*n* = 113). The dashed line is the mean of the difference between assay method results in Hp concentration (− 0.07 g/L) and the dotted lines are the mean ± 1.96 SD from the mean of this difference being 0.32 g/L and − 0.47 g/L respectively

In comparison to the previously established Hp-Hb binding assay, the novel IT assay gave a correlation coefficient (*R*^2^) of 0.95 with a regression equation of *y* = 0.98*x* + 0.09 (*y* = Hp in the IT assay; *x* = Hp by the Hp-HB binding assay), when the assays were performed on bovine samples (*n* = 108) with a range of low, medium and high concentrations of Hp (Fig. 5). These samples were selected so that they gave a reading in the Hb-Hp binding assay of above 0.03 g/L, which is the limit of detection of this assay. The same data plotted in a Bland-Altman plot showed a slight bias with the Hp-Hb giving a mean of 0.07 g/L less than the result of the IT assay (Fig. 6).

## Discussion

This IT assay for bovine Hp has been established, validated and can be employed for the rapid analysis of this major APP in cattle. Using a specific antiserum for bovine Hp, the analysis, performed on an automated biochemical analyser allows routine analysis of this biomarker of infection and inflammation to be incorporated into health assessment of both dairy and beef cattle. The assay has comparable levels of precision and accuracy to the Hp-Hb activity assay that has been used in laboratories based on the method first described by (Eckersall et al. 1999). The precision of the IT assay was 5% or less for intra assay CVs, and 8.1% or less for inter-assay CVs, comparable to the Hp-Hb binding assay. The accuracy was not determined in the previous Hp-Hb assay by determination of recovery, and so cannot be compared to the IT assay. However, both assays gave comparable results using parallel dilution curves. Specificity of the IT assay was demonstrated by the antibody cross-reactivity demonstrated by the Ouchterlony immunodiffusion gel. At present, the cross species reactivity of the antiserum has not been assessed, though as the antibody was raised in sheep, it is unlikely to react with ovine Hp. Interference by free Hb was evident at a concentration of 4 g/L with a 10% reduction but there was a lower interference than the Hp-Hb binding assay which a 17% reduction in Hp caused by free Hb in serum at a similar concentration. Samples with gross haemolysis should not be analysed with the IT Hp assay. Bilirubin and lipaemia had minimal effect on the IT Hp assay. Specificity by antibody cross-reactivity was only possible in the IT assay, which as it relies on the antibody specificity, was not possible in the Hp-Hb binding assay.

Definition of the LoQ for the IT assay is valuable for measuring Hp in bovine species as it has been long established that the Hp concentration in serum from healthy cattle is very low, indeed undetectable in some assays and would not be in the range for this IT analysis on a biochemical analyser. This was established in early investigations of bovine Hp (Young et al. 1995; Eckersall and Conner 1990) and can be seen in Fig. 1 of this report where there is no antibody reaction seen in the Ouchterlony gel where the sample is serum from a healthy cow. In this assay, the LoQ is 0.033 g/L and the reference range for Hp in serum from healthy animals is therefore < 0.033 g/L. Any result above, this would indicate the presence of an acute phase response. A more relevant figure would be a clinical decision point to aid interpretation and provide an indication when therapeutic intervention would be necessary. It would be useful if all assays for Hp, especially for species where the concentration in healthy animals is low or undetectable to have a well-defined LoQ and a clinical decision point. To define a clinical decision point is outwith the scope of this report and will require further trials with substantially more samples from cattle with clinical and sub-clinical disease. The replicates for LoQ were lower than ideal to conserve the antiserum reagent but do provide an indication of the sensitivity of the assay. If an IT assay were to be developed as a diagnostic kit, then this and the other validation parameters should be reassessed.

The detection and monitoring of sub-clinical disease using Hp will need assays with lower LoQ, such as those using the ELISA format, first noted in the literature over 20 years (Young et al. 1995) and more recently (Hiss et al. 2004; Cooke and Arthington 2013; Thomas et al. 2016). Although a limit of detection was reported in the earliest of these (Young et al. 1995), it was given in units of % Hb binding capacity (8 mg% HbBC) and not converted to grams per litre making comparison difficult to the LoQ for this IT assay. A LoD of 0.07 mg/L for an Hp ELISA, rather than LoQ, was reported by Hiss et al. (2004) which is 470 less than the LoQ of the IT assay demonstrating the greater sensitivity of the ELISA. When an ELISA was compared to a Hp-Hb assay for Hp in bovine samples, the ELISA measured a level of 0.11 μg/mL in serum, but a Hp-Hb binding assay was reported as giving a negative value of negative 10.35 μg/mL. This should have been reported as less than the LoD, but this was not defined (Cooke and Arthington 2013). The level of 0.11 μg/mL as reported is 300-fold less than the LoQ of the IT assay. However, the ELISA format is not suitable for most diagnostic laboratories while the IT method described here would be easily incorporated into a bovine health profile. Immunoturbidimetric assays can be adapted by using a latex enhanced approach and can reduce LoQ by around 10-fold. Whether this would be enough to allow monitoring of small changes in Hp concentration in assessment of low-level changes and whether this would be relevant to clinical diagnosis is a question that can be addressed by further development of a suitable high sensitivity assay.

Although IT assays were first described over 30 years ago (Price et al. 1983), there have been few recent reports of their development, despite the IT assay being the main method used for rapid measurement of high abundance proteins of serum in human clinical biochemistry laboratories. A valuable exception to this was the description of assays for 14 human serum proteins, including human Hp (Ledue et al. 2002), while our previously described IT assay for canine C-reactive protein (Eckersall et al. 1991) has recently led to commercial assays for veterinary diagnostic laboratories (Hillstrom et al. 2014; Hindenberg et al. 2017). Probably the main reason for limited description of IT assays in the literature is that most work on these methods is pursued by commercial diagnostic companies, who retain the assay details within the company for intellectual property reasons. It is known that IT assays can be run with antibody against a heterologous species, and IT assays for human proteins such as CRP are known to work for canine CRP (Klenner et al. 2010). It is possible that assays based on antiserum to human Hp would be effective in measuring this APP in bovine serum as has been reported for canine and equine serum (Tecles et al. 2007; Wiedmeyer and Solter 1996). However, it is always preferable to use a species-specific antiserum in IT assays, not least because changing batches of non-species specific antiserum can have major consequences on an established procedure.

While the early and low-level concentration of Hp in serum can already be detected by ELISA, this technology more suited to research projects than to the high-throughput clinical pathology laboratories where automation of analysis is important. However, the easy automation of the IT assay means that it can have wide applications in comparative clinical pathology laboratories. In species where Hp is present in measurable concentration in healthy animals such as dog, pig and human, the Hb-Hp binding assay can be used for this purpose, but the introduction of the automated IT assay will allow low-level change in Hp concentration to be assessed in cattle.

Direct comparison of this novel IT assay with the established Hp-Hb binding assay using 108 bovine serum samples using correlation and regression analyses showed that the IT assay gives equivalent results to that of the Hp-Hb assay, and it is therefore the recommendation of the authors that the IT assay gradually replace the previously accepted Hp-Hb assay. The Bland-Altman analysis revealed a minimal negative bias of 0.07 g/L. This bovine Hp assay has the potential to be included in bovine health assessment profiles, being in a format that enables inclusion in the workflow of diagnostic laboratories including use on analysers that are limited to two reagents. The IT assay could be able to detect the early stages of an acute phase reaction and such early changes are detectable throughout a herd, it could indicate the presence of low level of infection or inflammation that would allow early intervention at both individual and herd level to restore the health and the production of milk or meat.
